# External validation of the Baseline Recurrence Risk in Cellulitis (BRRISC) score and the added impact of acute clinical response: a prospective cohort study

**DOI:** 10.1186/s12879-025-12189-3

**Published:** 2025-12-29

**Authors:** Elizabeth L. A. Cross, Gail N. Hayward, Martin J. Llewelyn, A. Sarah Walker

**Affiliations:** 1 Department of Global Health and Infection, Brighton and Sussex Medical School, University of Brighton and University of Sussex, Brighton, BN1 9PX UK; 2 Department of Infection, University Hospitals Sussex NHS Foundation Trust, Eastern Road, Brighton, BN2 5BE UK; 3 Nuffield Department of Primary Care Health Sciences, University of Oxford, Radcliffe Primary Care Building, Radcliffe Observatory Quarter, Woodstock Rd, Oxford, OX2 6GG UK; 4 NIHR Healthtech Research Centre in Community Healthcare, Radcliffe Observatory Quarter, Woodstock Rd, Oxford, OX2 6GG UK; 5 Nuffield Department of Medicine, University of Oxford, John Radcliffe Hospital, Headley Way, Oxford, OX3 9DU UK; 6 NIHR Biomedical Research Centre, Oxford, OX3 9DU UK; 7 NIHR Health Protection Research Unit in Healthcare Associated Infections and Antimicrobial Resistance, University of Oxford, Oxford, OX3 9DU UK

**Keywords:** Cellulitis, Skin and soft tissue infection, Recurrence, Clinical response, Risk prediction

## Abstract

**Background:**

The BRRISC score was developed to predict hospital-attended cellulitis recurrence using clinical data routinely available at presentation. In practice, clinicians assess patients’ response to treatment during the recommended 48-72-hour antibiotic review point when deciding on the duration of antibiotic treatment to achieve lasting recovery. We evaluated the performance of the BRRISC score in an external validation cohort and examined whether incorporating markers of acute clinical response could improve it.

**Methods:**

We recruited adults with lower limb cellulitis attending hospital. From days 0–3 of treatment, we assessed markers of acute clinical response, including physical examination findings (e.g. affected size, oedema), objective measurements of limb temperature taken with a thermal imaging camera, vital signs, blood test results, and patient-reported symptoms. Outcomes included ‘hospital-attended recurrence’ (primary outcome for validation) and ‘any recurrence’ (hospital-attended or community). Using multivariate logistic regression with backwards elimination, we identified response variables independently associated with either outcome that could be included within an extended score. Performance was assessed using the C-index.

**Results:**

Of 202 patients, 8% (*n* = 17) experienced ‘hospital-attended recurrence’ and 23% (*n* = 46) ‘any recurrence’. In this validation dataset, the BRRISC score had a C-index = 0.75 (95%CI, 0.64–0.86) for predicting ‘hospital-attended recurrence’ vs. 0.65 (0.63–0.68) in the original development population, but only 0.60 (0.51–0.69) for the new outcome ‘any recurrence’. There was weak evidence that an extended score, incorporating day-2/3 severity of skin blistering, improved the C-index for ‘hospital-attended recurrence’ to 0.83 (0.75–0.92). No acute clinical response variables were independently associated with ‘any recurrence’ after adjusting for BRRISC score.

**Conclusions:**

The BRRISC score can help identify patients with cellulitis at the highest risk for hospital-attended recurrence. Markers of acute clinical response, typically used by clinicians to inform antibiotic treatment decisions during the 48-72-hour antibiotic review period, did not add helpful prognostic value beyond the baseline factors included in the score. Whether treatment response after day-3 improves recurrence prediction could be further explored, but future research should focus on evaluating the potential for baseline risk stratification to personalise antibiotic duration and guide non-antibiotic approaches for preventing recurrence.

**Clinical trial number:**

Not applicable.

**Supplementary Information:**

The online version contains supplementary material available at 10.1186/s12879-025-12189-3.

## Background

Cellulitis is a common and debilitating skin infection [1]. Although most patients recover clinically in the short-term, 15–50% experience recurrence in the months following an initial episode [2–5]. Treatment guidelines for cellulitis recommend 5–7 days of antibiotics [6, 7], but evidence demonstrates that most patients are treated for considerably longer [8–10]. Clinicians may prolong therapy to try to avoid poor clinical outcomes, but whether prolonged antibiotic treatment reduces recurrence risk in some patients is unclear [4]. 

Two small, retrospective studies have previously attempted to develop risk scores to identify patients at high risk of recurrence [2, 11]. While these scores demonstrated good performance within their respective development cohorts (0.72 and 0.78), neither has undergone successful external validation. Moreover, both previous scores excluded patients with previous cellulitis (18% of otherwise eligible patients in one study),^11^ limiting relevance to clinical practice. Subsequently, we developed the BRRISC score to predict hospital-attended cellulitis recurrence [12]. The score was derived using electronic health records (EHRs) containing clinical data available at baseline (age, heart rate, urea, platelet count, albumin) and comorbidities (previous cellulitis, venous insufficiency, liver disease). While recurrence rates increased fourfold across risk groups, score performance was only moderate (C-index = 0.65 (95% CI, 0.63–0.68)). We hypothesised that this might reflect a lack of data on clinical response, which clinicians use in everyday practice when deciding antibiotic therapy duration [13, 14]. Further, no external dataset was available for validation.

Here, we followed a cohort of UK adults with lower limb cellulitis to capture early clinical response and recurrences. Our objectives were to externally validate the BRRISC score and determine whether adding acute clinical response would improve prognostic value.

## Methods

### Population and setting

Patients were recruited from two acute hospitals in the UK’s National Health Service (NHS) from June 2021 to March 2023. Adults (≥ 18 years) were eligible if their treating clinician identified them as having lower limb cellulitis requiring antibiotic treatment. The main exclusion criteria were already receiving ≥ 3 calendar days of antibiotics from the hospital for cellulitis, being treated for a previous cellulitis episode in the preceding 28 days, or if the clinical diagnosis changed to an alternative diagnosis within three days of enrolment (Figure S1; details in Appendix p1). See Appendix p2-3 for sample size calculation.

### Study assessments

Data was collected on demographics, previous admissions (including diagnosis codes), and microbiological results. Previous cellulitis was defined in two ways. The first, ‘patient-reported’ was self-reported by patients. The second, ‘hospital-attended previous cellulitis’, replicated the definition used in the BRRISC score development and was based on any previous hospital attendance containing a cellulitis diagnosis code in any position identified from EHRs (Appendix p3).

Acute clinical responses were assessed by physical examination (including the use of a Cellulitis Severity Score (CSS) [4, 15], Table S1), limb temperature measurements, vital signs, blood test results (infection markers (C-reactive protein (CRP), neutrophil count), tests from the BRRISC score (platelets, urea, albumin)), and patient-reported pain and swelling due to cellulitis. Where possible, physical examination was performed on all patients daily for four days beginning on day 0, defined as the date the patient began their hospital-initiated antibiotic treatment for cellulitis. For further details on acute clinical response variables, see Appendix p3. We used mixed models to estimate changes in these parameters over time, assuming data were missing at random (correlated patient-specific intercept and slope).

Antibiotic data to calculate length of therapy (LOT; duration) and days of therapy (DOT; aggregate sum of days of each agent received) were collected from day 0 to the completion of hospital-initiated antibiotic treatment. Primary care records were also reviewed to determine if hospital-initiated antibiotic therapy was continued in the community and contributed to the calculated LOT and DOT. Community-prescribed antibiotics received prior to initial hospital attendance were not included in the calculation of LOT and DOT. Antibiotic data for the development cohort consisted of hospital-prescribed antibiotics, including discharge medications, but no community antibiotic use.

### Outcomes

The primary outcome for the cohort was ‘any recurrence’ within 90 days, defined as initiating new antibiotic treatment in primary care or hospital for cellulitis at the same site. Recurrence was assessed at a follow-up visit or if the patient was uncontactable via EHRs. The BRRISC score was originally developed using the narrower definition of ‘hospital-attended recurrence’ within 90 days, so this was also considered for primary score validation (Appendix p3).

### Statistical analysis

For missing data in acute clinical responses, we considered both hard imputations (day 0/1 = day-0 otherwise day-1; day 2/3 = day-3 otherwise day-2) and performed multiple imputation of variables in Table S2 by chained equations with predictive mean matching (missing at random assumption; 50 imputed datasets). Imputed and observed values were compared visually (Figures S2 & S3).

We first externally validated the BRRISC score’s ability to predict ‘hospital-attended recurrence’. Patients who died within 90 days without experiencing a recurrence were excluded from the analysis (effectively considering the first two components of a multinomial outcome; no recurrence/death, recurrence before death, death before recurrence), as was done for original score development [12]. Mortality among patients with cellulitis is relatively low, even among hospitalised patients, and only a minority of deaths are thought to be directly caused by the infection itself [16]. Therefore, as patients with cellulitis are often older adults with multiple co-morbidities receiving hospital-based care, the underlying risk factors for death are likely to differ from those for recurrence, and combining the two could obscure associations. We subsequently tested the score’s ability to predict the new outcome, ‘any recurrence’. Performance was measured using Harrell’s C-index/area under the ROC curve in the imputed datasets [17, 18]. For each BRRISC score level, mean observed risks across imputed datasets were compared versus predicted risks from the original model. Similarly to the outcomes, the BRRISC score variable ‘previous cellulitis’ can be defined as ‘patient-reported’ or ‘hospital-attended’, so the above steps were performed for each definition, leading to four separate prediction models.

To identify whether acute clinical response could improve BRRISC score performance, we considered all additional variables with univariate *p* < 0.05 for either recurrence outcome (details in Table S3) alongside the BRRISC score in multivariate logistic regression models, using backwards elimination on non-BRRISC score variables to identify independent predictors, and using fractional polynomials to allow for non-linearity in continuous factors. We compared the C-indexes from the original and extended scores in each imputed dataset [19], and assessed the contribution of new predictors using a category-based Net Reclassification Index (NRI) [12]. For further details on score validation and extension, see Appendix p4.

Stata v18.0 (StataCorp LLC, College Station, TX) was used for all analyses. We followed the Transparent Reporting of a multivariable prediction model for Individual Prognosis Or Diagnosis (TRIPOD) statement (Table S4) [20]. 

### Public and patient involvement

This study involved patients and the public in the design and conduct of the research through the James Lind Alliance Cellulitis Priority Setting Partnership [21] and a group consisting of people with lived experience of cellulitis. Our contributors checked the acceptability of the study procedures, edited patient information materials, and improved outcome measure collection.

## Results

202 patients with unilateral lower limb cellulitis were included (Figure S1); the median age was 66 years (interquartile range, IQR 51,79), 84 (42%) were female, and 191 (95%) of white ethnicity. Over half (105, 52%) self-reported a previous cellulitis episode, with 55 (27%) experiencing a previous episode requiring hospital attendance, higher than that of the development cohort (5%) (patient characteristics in Table S5).

### Acute clinical response

**Fig. 1:**
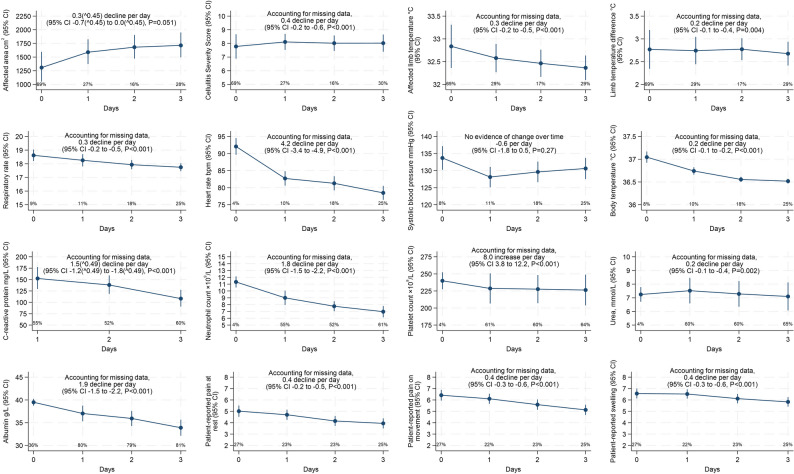
Acute clinical responses over first four days from initiation of antibiotics (Day-0). Note: Day-0 was taken as the date of hospital antibiotic initiation. Percentages indicate missing data per day. Points show observed data. Estimates of change over time from linear mixed models with correlated patient-specific intercept and slope which allow for missing data at random (which includes missing data depending on previous observed values). Affected area and CRP stated declines are transformed values. CSS (Cellulitis Severity Score): Made up of 7 components, each scored 0–3, higher numbers indicate more severe findings, see Table S1. Patient-reported symptoms scored 0–10, higher numbers indicate more severe symptoms

Over days 0–3, total CSS, affected limb temperature, respiratory rate, heart rate, body temperature, CRP, neutrophil count, urea, albumin, and patient-reported symptoms decreased (*P* < 0.001), whilst platelet count increased (*P* < 0.001) (observed data and results from linear mixed models Fig. 1). There was moderate evidence for decreases in limb temperature difference (*P* = 0.04), weak evidence for decreases in affected skin area (*P* = 0.051), and no evidence for changes in systolic blood pressure (*P* = 0.27).

**Fig. 2:**
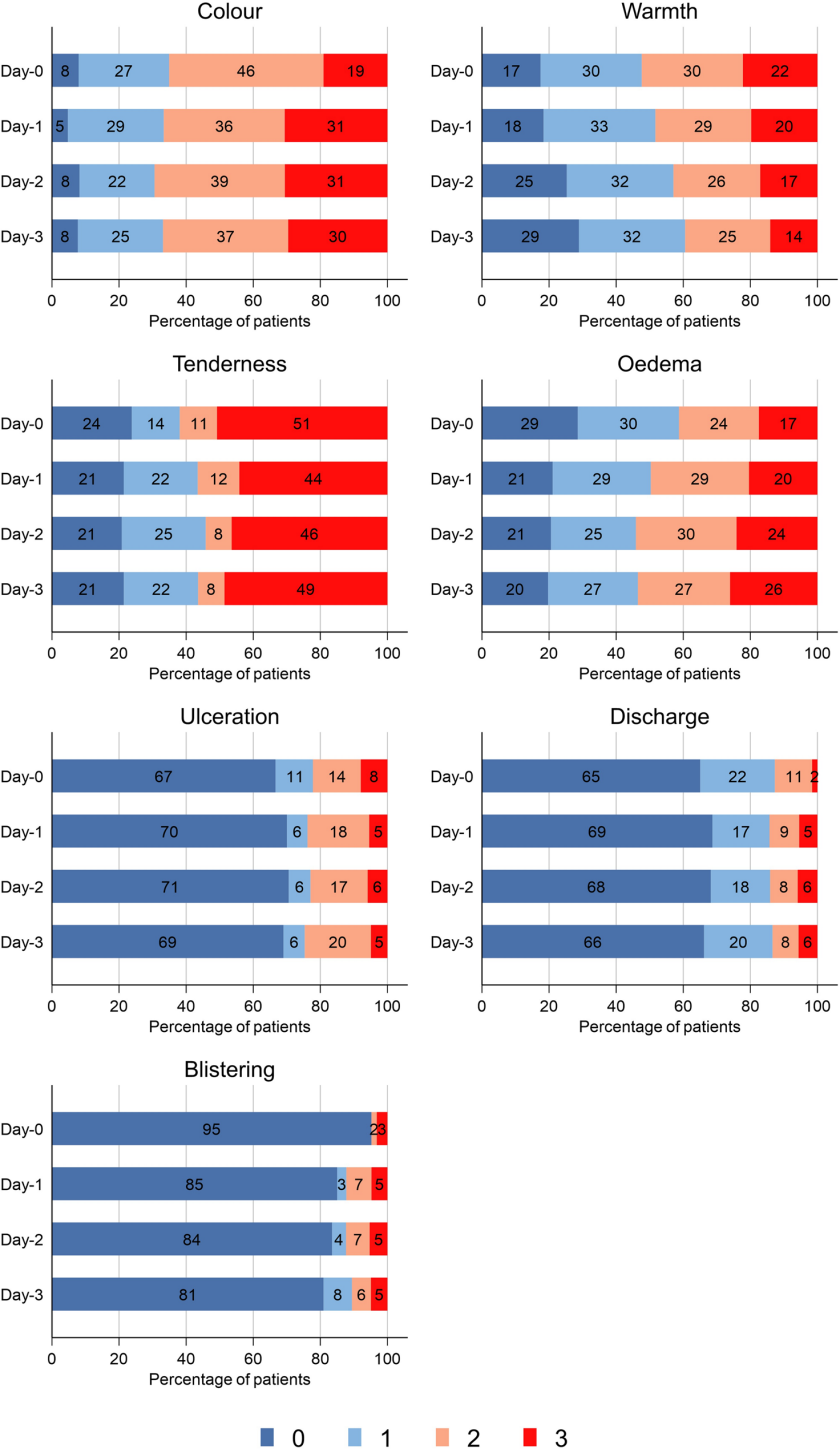
Cellulitis Severity Score (CSS) subcomponents over first four days from initiation of antibiotics (Day-0). Note: Day-0 was taken as the date of hospital antibiotic initiation. The missing data for days 0–3 were 69%, 27%, 16%, and 30%, respectively

In terms of CSS subcomponents, the odds of higher colour, warmth, and oedema subscore decreased per day by 53% (95%CI 0.34–0.64, *P* < 0.001), 53% (0.37–0.60, *P* < 0.001), and 27% (0.55–0.98, *P* = 0.04), respectively (Fig. 2). The odds of a higher blistering subscore increased per day by 96% (1.07–3.63, *P* = 0.03). There was no evidence for a change in the tenderness subscore over time (OR = 0.86, 0.67–1.10, *P* = 0.24) (models for ulceration and discharge subscores did not converge).

### Antibiotic use

Before hospital attendance, 61 (30%) patients received a median 3 days (IQR 2,4) oral antibiotic therapy from the community. The most common hospital-prescribed antibiotics were flucloxacillin (85,42%), ceftriaxone (52,26%), and clindamycin (28,14%). The median antibiotic LOT/duration was 13 days (IQR 10,18), and DOT was 15 days (IQR 10,22) (details in Table S5). Of note, two patients (1%) were prescribed prophylactic antibiotics for cellulitis pre-enrolment.

### Outcomes

The primary study outcome, ‘any recurrence’ within 90 days, occurred in 46 (23%) patients, a median of 31 (IQR 22,56) days from initial hospital attendance (Figure S4). ‘Hospital-attended recurrence’ (primary outcome for BRRISC validation) occurred in 17 (8%) patients, a median of 37 (IQR 24,66) days from initial hospital attendance.

### BRRISC score external validation and test

The BRRISC score had a C-index of 0.75 (95%CI, 0.64–0.86) for predicting ‘hospital-attended recurrence’, regardless of the definition of previous cellulitis used (Table 1) vs. 0.65 (0.63–0.68) in the original development population based on EHRs [12]. When tested on the new outcome ‘any recurrence’, the C-index was 0.60 (0.51–0.69) and 0.63 (0.54–0.71) for ‘patient-reported’ or ‘hospital-attended’ definitions of previous cellulitis, respectively.

**Table 1:** Model performance

Definition of previous cellulitis	Variable	Beta Coefficient (per unit higher)	OR	95%CI	*P*	AUC	95%CI
**External validation of BRRISC score (original score and outcome (hospital-attended recurrence))**							
Patient-reported	BRRISC score	0.43	1.54	1.19-2.00	0.001	0.75	0.65–0.85
Hospital-attended	BRRISC score	0.42	1.52	1.19–1.94	0.001	0.75*	0.64–0.86
**Test of BRRISC score with new outcome (any recurrence)**							
Patient-reported	BRRISC score	0.17	1.19	1.00-1.41	0.04	0.60	0.51–0.69
Hospital-attended	BRRISC score	0.21	1.24	1.04–1.46	0.01	0.63	0.54–0.71
**Model incl. BRRISC score and day-2/3 blistering (original outcome (hospital-attended recurrence))** ^†^							
Patient-reported	BRRISC score	0.52	1.68	1.25–2.26	0.001	0.84	0.75–0.92
	Day-2/3 blistering	0.97	2.63	1.56–4.43	< 0.001		
Hospital-attended	BRRISC score	0.50	1.65	1.24–2.20	0.001	0.82	0.72–0.93
	Day-2/3 blistering	0.95	2.58	1.55–4.29	< 0.001		
**Extended score (incorporating day-2/3 blistering (original outcome (hospital-attended recurrence))**							
Patient-reported	Extended score	0.40	1.49	1.24–1.79	< 0.001	0.83	0.75–0.92
Hospital-attended	Extended score	0.39	1.48	1.24–1.76	< 0.001	0.82	0.72–0.92

Note: In development dataset, using hospital-attended previous cellulitis and hospital-attended recurrence outcome, AUC = 0.65 (95% CI, 0.63–0.68) (* indicates validation equivalent) [11].

^†^Assigning 2, 4, and 8 points for day-2/3 blistering subscores of 1, 2, and 3, respectively, based on beta coefficients from the combined model considering day-2/3 blistering linearly or categorically (Table S6) increased the maximum possible score from 15 to 23 points. Tests of equality of the BRRISC and extended score C-indexes in each imputed dataset indicated weak evidence that the extended score C-index was higher than the BRRISC score (Figures S5 & 6)

**Fig. 3:**
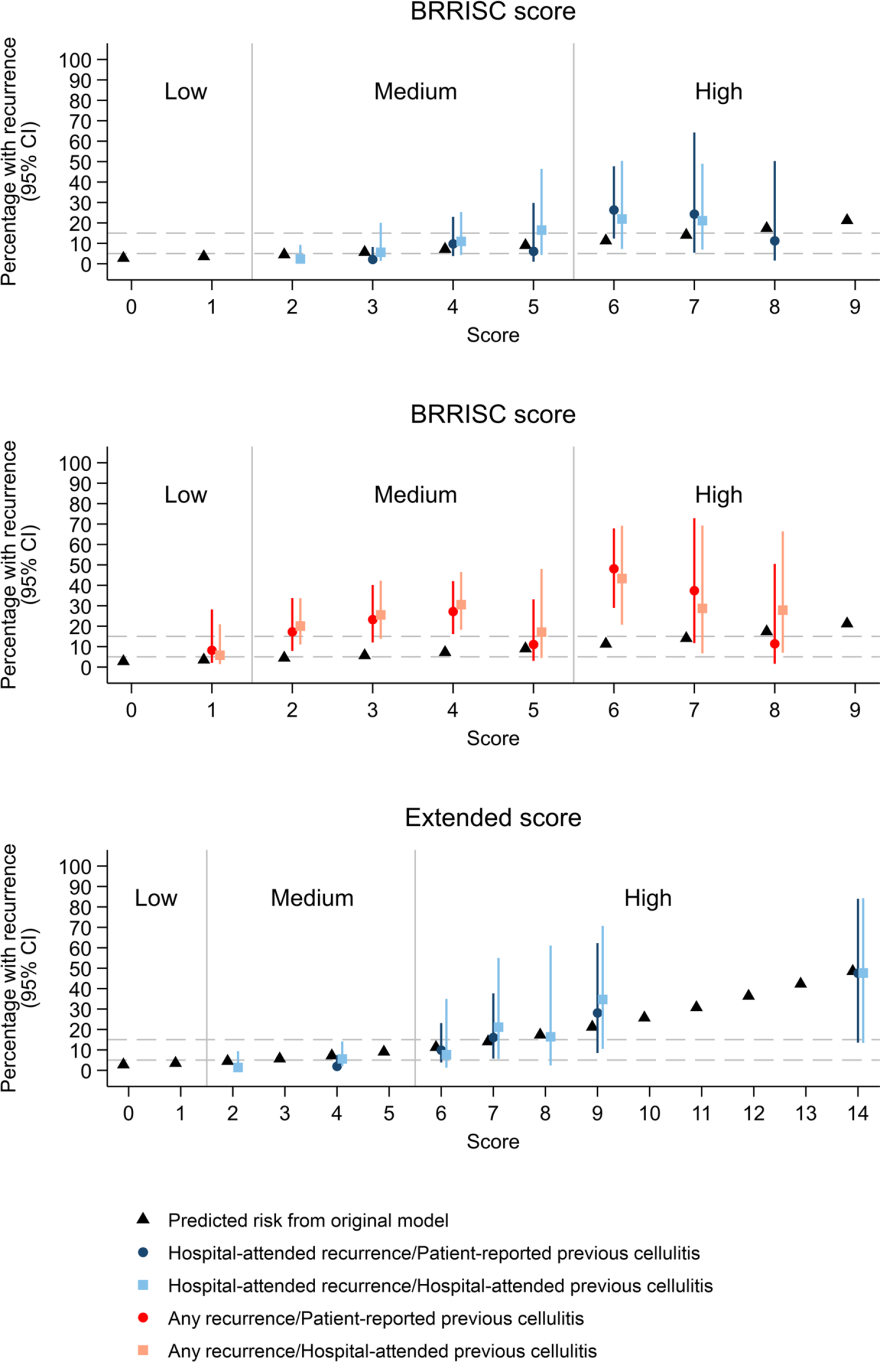
Observed (95%CI) and predicted percentage with recurrence per score level. Note: As some imputations contained zero recurrences in one or more of the score levels, observed risks were combined across some score levels to calculate estimated observed risk across imputed datasets (BRRISC score dark blue 3 = 0–3, 8 = 8–9, light blue 2 = 0–2, 7 = 7–9, red and pink 1 = 0–1, 8 = 8–9; Extended score dark blue 4 = 0–4, 6 = 5–6 7 = 7–8, 9 = 9–11, 14 = 12–14 & 17, light blue 2 = 0–2, 4 = 3–5, 9 = 9–11, 14 = 12–14 & 17). Previously defined risk thresholds at 5% and 15% categorised scores as low (score 0–1), medium (2–5) and high (6–15) risk. Table S7 for Net Reclassification Index relating to extended score

The percentages with recurrence by either previous cellulitis definition generally increased with increasing BRRISC score up to a score of 6, with wide 95% CI above this (Fig. 3). For the ‘hospital-attended recurrence’ outcome, on which the score was developed, 95% CIs generally overlapped the risk predictions from the original model, whereas risk estimates were generally higher for the ‘any recurrence’ outcome, as expected.

### BRRISC score extension using acute clinical response

Of the acute clinical response variables univariately associated with ‘hospital-attended recurrence’ (Table S3), only day-2/3 skin blistering (part of the CSS, see Table S1) remained independently associated after adjusting for the BRRISC score, with C-indexes 0.84 (95%CI, 0.75–0.92) and 0.82 (0.72–0.93) for ‘patient-reported’ and ‘hospital-attended’ previous cellulitis definitions, respectively (Table 1). C-indexes were similar for an extended score incorporating day-2/3 skin blistering (Table 1; Fig. 3**)**. Tests of equality of the BRRISC and extended score C-indexes in each imputed dataset indicated weak evidence that the extended score C-index was higher than the BRRISC score (Figures S5 & 6).

Using the extended BRRISC score, the reclassification of imputations into risk groups produced an event NRI of + 34%/+16% (correctly reclassified high-risk patients) and non-event NRI of -12%/-3% (more often incorrectly classified low-risk patients as high-risk), using ‘patient-reported’/’hospital-attended’ previous cellulitis, respectively (Table S7). The summary NRIs were, therefore, + 22% and + 13%, respectively.

As none of the variables univariately associated with the new ‘any recurrence’ outcome at *p* < 0.15 (Table S3) remained associated with the outcome at *p* < 0.05 after adjusting for the BRRISC score, we did not consider extending the BRRISC score to predict ‘any recurrence’.

## Discussion

The BRRISC score demonstrated good predictive performance for hospital-attended cellulitis recurrence in an external prospective cohort of UK adults [22]. Patients showed improvements in most markers of acute clinical response during the first few days of treatment. However, we found, at most, weak evidence that incorporating day-2/3 blistering improved the score’s performance, a finding that may be due to chance and should be interpreted with caution. Furthermore, extending the score to include day-2/3 blistering also more often incorrectly classified low-risk patients as high-risk. Taken together, these findings support continued use of the original BRRISC score alone, without the inclusion of day-2/3 blistering. Notably, the extent of day-2/3 blistering also more likely reflects the severity of the initial illness rather than a true indicator of treatment response. Of note, none of the acute clinical response variables independently predicted ‘any recurrence’ after adjusting for the BRRISC score.

A key difference between this cohort and the development sample is the higher proportion of patients with a previous episode of cellulitis requiring hospital attendance (27% vs. 5%). Additionally, over half of the patients in this cohort self-reported ever having a previous cellulitis episode. However, score performance remained consistent, regardless of how previous cellulitis was defined. This demonstrates that a broader, patient-reported definition (rather than only prior hospital-attended cases) can be reliably applied.

Other studies assessing early treatment response in cellulitis have reported substantial improvements within the first three days of treatment. Montalto et al. monitored limb temperature over time and found significant decreases in temperature change between the affected and unaffected limbs during the first 3 days of treatment, but no evidence for decreases from day-4 until day-7, although the sample size was smaller during this period [23]. In a pilot study testing an algorithm for intravenous to oral switch in 128 hospitalised patients with cellulitis, just over three-quarters showed significant clinical improvement within 48 h of treatment [10]. Similarly, among 216 patients with cellulitis, Bruun et al. found 90% had a measurable improvement in local inflammation and CRP by day-3 of treatment, and the strongest concordance between clinical and biochemical response occurred on days-2/3 [13]. As in our study, day-3 response did not predict a poor outcome (clinical failure).

A systematic review and meta-analysis of antibiotic trials for cellulitis with data on treatment response found the time to clinical response overall was 1.68 days (95%CI, 1.48–1.88) [24]. However, time courses varied between parameters. For example, the proportion of patients with oedema fell 30–50% by day 2–4 and there was a ~ 50% reduction in pain and severity scores by day-5. The authors concluded that the optimal clinical reassessment time is likely between 2 and 4 days, but this was based on four heterogeneous studies.

Unsurprisingly, some evidence does suggest continued improvement beyond day-3. Willliams et al., analysing data from the adjunctive clindamycin for cellulitis trial, found very strong evidence for a decrease in limb circumference and temperature difference by day-5 (median 4 days follow-up), though approximately one-third of patients still exhibited residual signs by day 10 (e.g., ≥ 2 cm limb circumference difference or ≥ 1 °C temperature difference) [25]. Similarly, a recent Australian prospective cohort study of 300 patients with cellulitis reported the greatest clinical improvement by day-3, with slower ongoing improvement at days 7 and 14. No independent predictors of cure/recovery were identified [26]. 

A limitation of our study is that by only assessing response up to day-3, we could have missed the potential prognostic value of assessment after that timepoint. However, the existing evidence supports that the most substantial improvements in clinical response occur within 48–72 h. This aligns with current guidelines recommending antibiotic treatment review within this timeframe [7, 27, 28], as well as the US Food and Drug Administration’s guidance, which uses early response at 48–72 h as the primary outcome measure in antibiotic licensing trials for cellulitis [29]. 

A further limitation is that we used a relatively crude method for measuring change in the size of affected skin area. This could explain why we found only weak evidence for a decrease despite a reduction in lesion size at 48–72 h being the recommended primary efficacy endpoint for cellulitis antibiotic trials [29]. Similarly, we measured skin temperature by locating the hottest point on the limb, but more advanced techniques that capture changes in the area of increased temperature are emerging [30]. Nonetheless, the approach we used was selected because it exemplifies the sort of approach clinicians could realistically apply in everyday practice.

Nearly one-third of the patients in our study received antibiotics before admission, consistent with observational studies in similar settings. Assuming some of these patients were presenting with partially treated infection rather than treatment failure,^9^ this could have diluted our ability to detect an association between response to treatment after study day-0 and outcome. Our pragmatic inclusion criteria of ‘clinician-treated cellulitis’ is another source of potential dilutional bias, as misdiagnosis rates have been found to be as high as 40% [31, 32]. If some patients did not have true cellulitis, they would not be expected to respond to antibiotic treatment. However, we excluded patients if their clinical diagnosis changed within three days of enrolment or if the investigator judged they did not have a clear diagnosis of cellulitis.

Other limitations of this study relate to generalisability. We only included patients from two hospitals, although they represented a mixture of tertiary care and district general services. Our findings do not necessarily apply to cellulitis affecting other body parts, less severe cases managed in the community, and/or patients treated with only oral therapy (only 6.4% of our cohort).

In addition, most patients were of white ethnicity, consistent with most of the published research in this area. Clinical response assessments that rely heavily on erythema may be less applicable to individuals with darker skin tones, in whom such changes may be less easily detected. We used the term ‘acute colour change’ in place of erythema, and measured lesion size based on the largest extent of either colour change, oedema, or induration. Nevertheless, the broader applicability of these assessment methods in more diverse populations remains to be established.

A key strength of our study was the use of an objective method for monitoring limb temperature over time, which only three studies have previously attempted [23, 25, 30]. Future research should explore whether temperature-based assessments, such as thermal imaging, might prove more valuable than measures of colour change in individuals with darker skin tones.

Another notable strength was our extended follow-up period. We observed patients until day 90, while most previous studies have only followed patients up to ≤ 30 days [33]. Of note, approximately half of the recurrences in this cohort occurred after day 30, highlighting a substantial burden of disease beyond the first month (Figure S4). Finally, our findings add to the growing body of evidence that most patients with cellulitis are treated for considerably longer than guidelines recommend [8–10]. 

## Conclusions

We successfully externally validated the BRRISC score in a new patient population, confirming that patient predisposition and illness severity at presentation can identify those at highest risk of hospital-attended cellulitis recurrence. To date, no other study has comprehensively examined the prognostic value of such a wide range of acute clinical response variables on clinical outcome in cellulitis. Future research could investigate whether treatment response between days 4 and 7 provides additional prognostic value, as this period remains clinically relevant for decisions about antibiotic duration. However, existing evidence suggests that symptom improvement beyond day-3 typically occurs more gradually. Taken together with our findings, this supports that clinicians can reasonably estimate recurrence risk on baseline characteristics alone.

Randomised controlled trials are needed to determine whether recurrence risk can guide antibiotic treatment options for cellulitis, and if responses after day-3 were predictive, this may necessitate consideration of longer antibiotic durations than the current 5–7 days for at least some patients. However, antibiotic strategies alone are unlikely to address the burden of recurrent cellulitis sufficiently. Greater attention must also be given to non-antibiotic approaches, such as compression therapy and enhanced skin care, particularly for patients at high risk of recurrence. These approaches are supported by guidelines yet remain underused and under-evaluated [5]. Future research should determine both their effectiveness and uptake in routine care.

## Supplementary Information

Below is the link to the electronic supplementary material.

Supplementary Material 1

## Data Availability

The datasets used and/or analysed during the current study are available from the corresponding author on reasonable request.

## Abbreviations

BRRISC: Baseline Recurrence Risk in Cellulitis
DOT: Days of therapy
EHR: Electronic health record
LOT: Length of therapy
NHS: National Health Service
CSS: Cellulitis Severity Score
CRP: C-reactive protein
NRI: Net Reclassification Index
